# Correction to “Natural Gas and Biogenic CH_4_ Emissions from an Urban Center, Sakai, Japan, Based on Simultaneous Measurements of CH_4_ and C_2_H_6_ fluxes Based on the Eddy Covariance Method”

**DOI:** 10.1021/acs.est.6c01933

**Published:** 2026-02-26

**Authors:** Masahito Ueyama, Akira Nakaoka, Taku Umezawa, Yukio Terao, Mark Lunt

This article included an error in the calculation of daily CH_4_ fluxes. Specifically, an incorrect treatment of bootstrap-derived statistics was applied when aggregating half-hourly fluxes to daily values, which led to an underestimation of daily CH_4_ fluxes and the resulting annual emissions.

The daily and annual CH_4_ fluxes have been recalculated using an appropriate temporal integration of half-hourly fluxes. The revised statistical values are summarized in Table 1. As a consequence, Figures 5 and 6 and Figures [Fig figS5] and [Fig figS6] in the Supporting Information have been updated and appear below.

**Figure 5:**
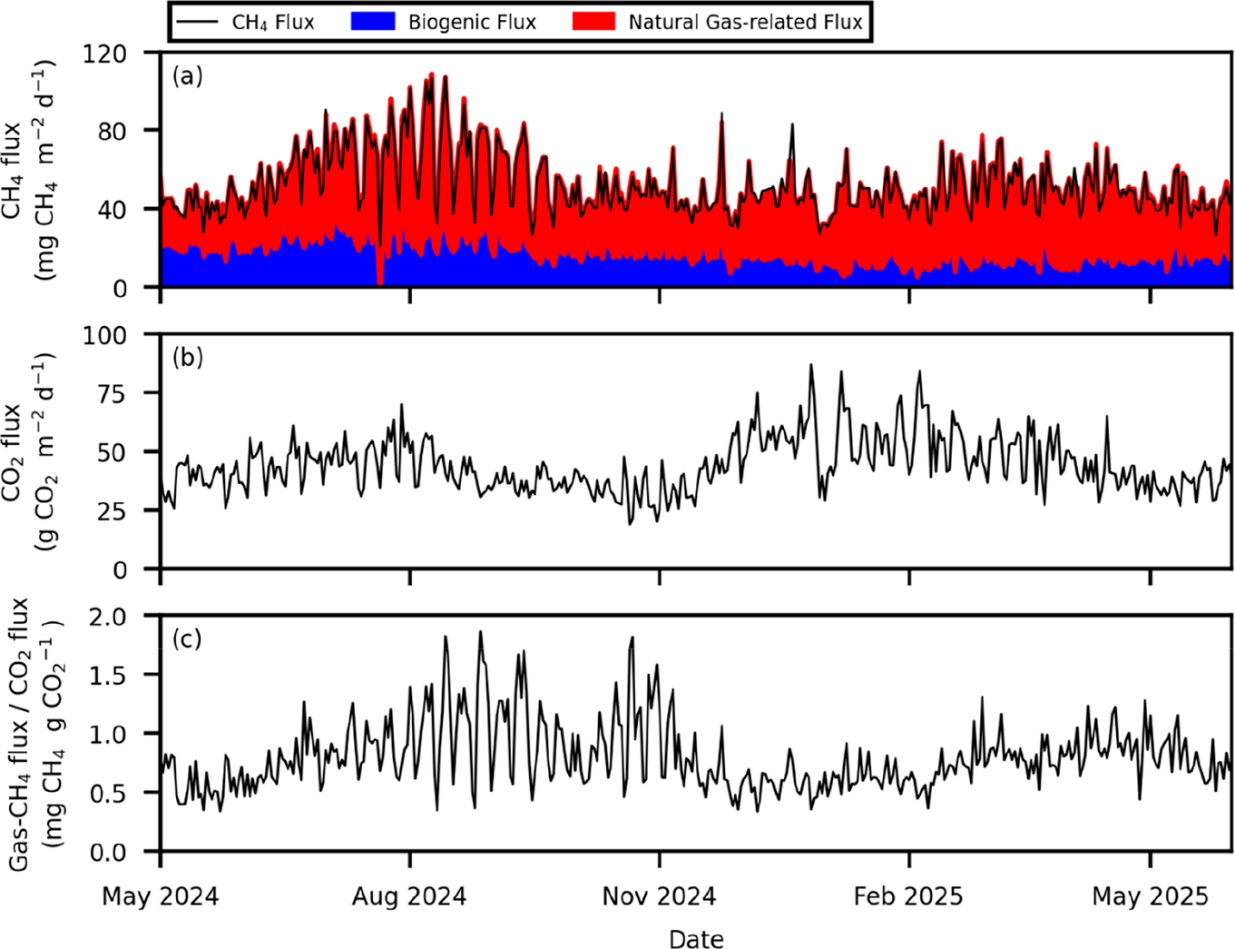
Daily variations of CH_4_ flux (a), CO_2_ flux (b), and the ratio of natural gas-related CH_4_ flux to CO_2_ flux (c). Note that both the partitioned CH_4_ flux (without stacking) and the total CH_4_ flux (line) are gap-filled individually; therefore, the two may show slight differences.

**Table 1:** Annual Fluxes of CH_4_ and CO_2_, Including the Partitioned Natural Gas-Related and Biogenic CH_4_ Components, and the Resulting Greenhouse Gas (GHG) Fluxes Expressed as CO_2_ Equivalents under 100-Year (GWP_100_) and 20-Year (GWP_20_) Global Warming Potential Metrics

flux	value
CH_4_ flux	19.2 g of CH_4_ m^–2^ year^–1^
CH_4_ flux gas	12.8 g of CH_4_ m^–2^ year^–1^
CH_4_ flux biogenic	6.6 g of CH_4_ m^–2^ year^–1^
CO_2_ flux	16.3 kg of CO_2_ m^–2^ year^–1^
GHG flux under GWP_100_	16.8 kg of CO_2_-equiv m^–2^ year^–1^
GHG flux under GWP_20_	17.8 kg of CO_2_-equiv m^–2^ year^–1^

**Figure 6:**
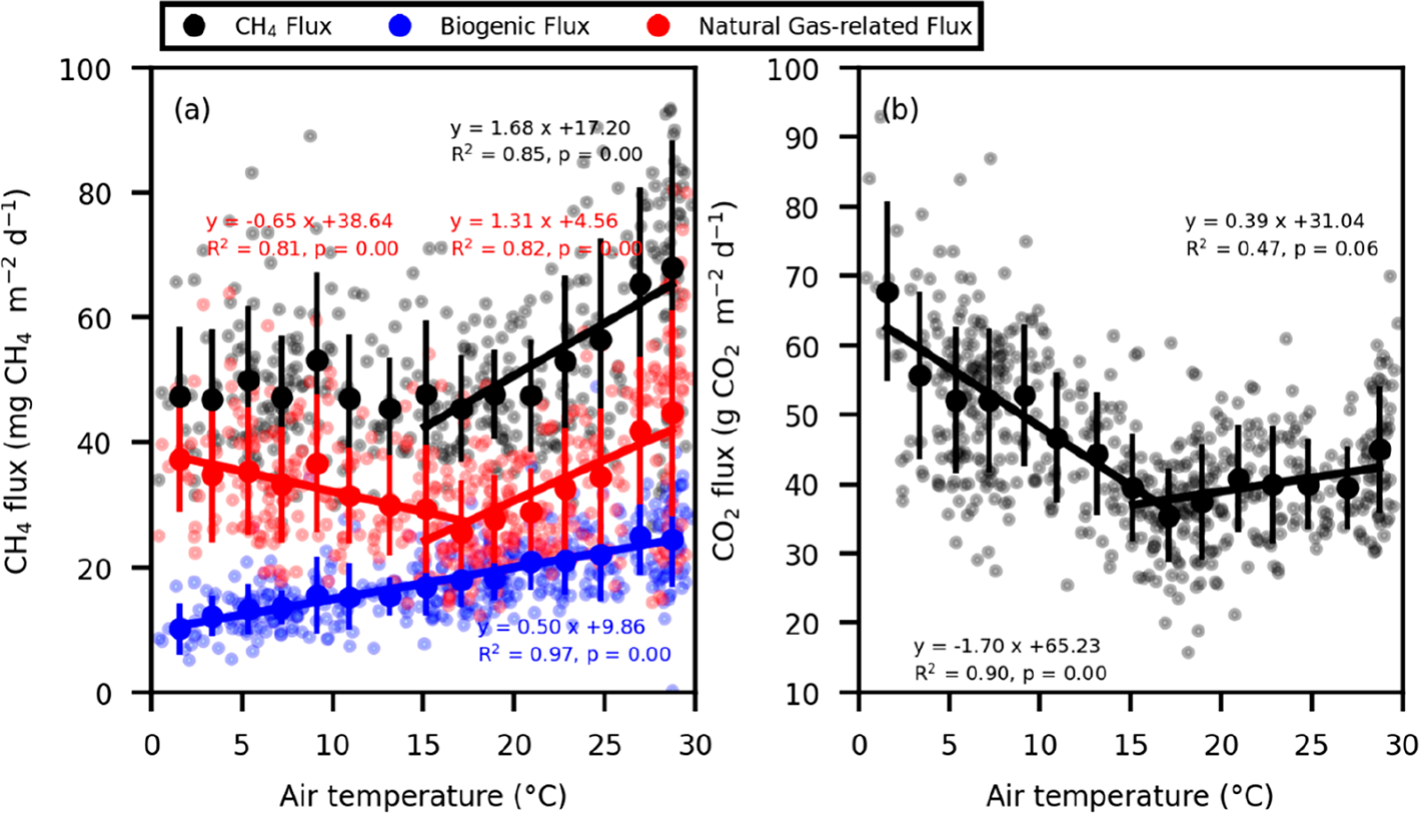
Relationships between daily mean air temperature and daily mean values of CH_4_ fluxes, partitioned between biogenic and natural gas-related fluxes (a) and CO_2_ fluxes (b).

**S5 figS5:**
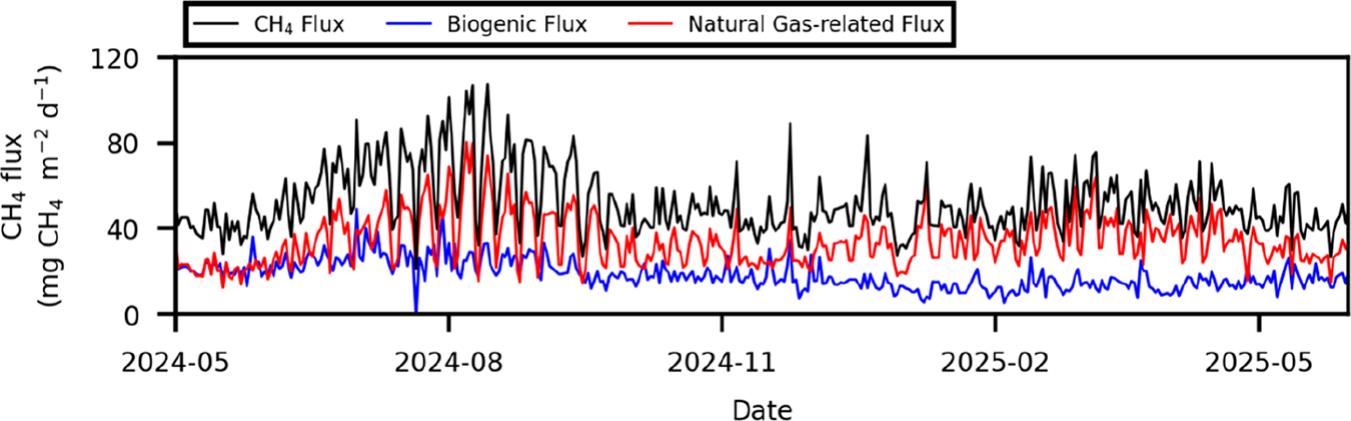
Daily variations of CH_4_ flux, natural gas-related CH_4_ flux, and biogenic CH_4_ flux. The figure presents the same data as the stacked bar chart in Figure 5a, but shown in a nonstacked format.

**S6 figS6:**
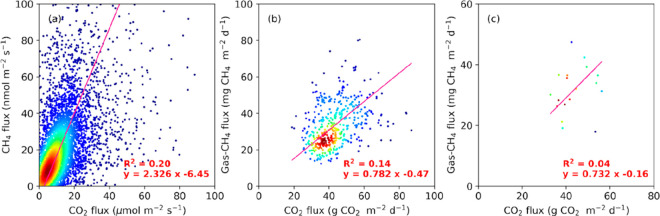
Density plots between CO_2_ flux and natural gas-related CH_4_ flux at half-hourly (a), daily (b), and monthly (c) time scales. The pink line represents the total least-squares regression.

The correct annual CH_4_ emission is 19.2 g of CH_4_ m^–2^ year^–1^, which is approximately twice the originally reported value. This correction does not affect the seasonal characteristics of CH_4_ emissions, the relative importance of biogenic and natural gas-related sources, or the main conclusions of the study.

